# Changes in Tobacco and Alcohol Consumption in France during the Spring 2020 Lockdown: Results of the Coviprev and Viquop Surveys

**DOI:** 10.3390/ijerph192214808

**Published:** 2022-11-10

**Authors:** Guillemette Quatremère, Romain Guignard, Raphaël Andler, Sandie Sempé, Nathalie Houzelle, Viêt Nguyen-Thanh

**Affiliations:** 1 Addictions Unit, Department for Preventive Healthcare and Health Promotion, Santé Publique France, The French National Public Health Agency, 12 rue du Val d’Osne, Allée Vacassy, 94410 Saint-Maurice, France; 2 Health Promotion, Perinatal and Early Childhood Unit, Department for Preventive Healthcare and Health Promotion, Santé Publique France, The French National Public Health Agency, 12 rue du Val d’Osne, Allée Vacassy, 94410 Saint-Maurice, France

**Keywords:** COVID-19, SARS-CoV-2, alcohol, tobacco, lockdown

## Abstract

This study aims to describe changes in tobacco and alcohol consumption in France during the first COVID-19 lockdown in March 2020 and its gradual lifting in May. The associated factors and the reasons reported explaining those changes are also studied. Data came from five waves of the CoviPrev online cross-sectional survey (approximately n = 2000 per wave) and the ViQuoP qualitative survey (n = 60), which took place between April and June. Most people self-reported stable consumption compared to before the lockdown, but 27% to 32% of smokers and 10% to 16% of drinkers had increased their consumption, depending on the wave of the survey. Boredom, stress and the search for pleasure were the main reasons reported. While the sociodemographic factors associated with an increase in tobacco and alcohol use differed according to the product and month, poor mental health was associated with an increase in both products in April and May. Between 10% and 19% of smokers and 22% to 25% of drinkers reported having reduced their consumption for their health or through constraints. The measures taken to manage the spring 2020 epidemic appear to have had contrasting impacts on tobacco and alcohol consumption in France. People whose lifestyles and mental health was most affected appear to have modified their consumption more frequently.

## 1. Introduction

Tobacco and alcohol are two major health determinants worldwide [1,2]. In France, they are responsible for 75,000 and 41,000 annual deaths, respectively [3,4]. The scientific literature shows that the use of psychoactive substances, including tobacco and alcohol, is associated with poor mental health [5,6,7,8,9,10]. The onset of the COVID-19 epidemic and the resulting national lockdown measures implemented from 17 March 2020–lifted gradually from 11 May 2020–impacted the mental health of the French population, increasing levels of stress, anxiety and depression, particularly for people with a low socioeconomic level and women [11,12]. The context of the epidemic may therefore have had a negative impact on tobacco and alcohol consumption.

In addition, the travel restrictions, the increase in working from home, the closures of schools and some shops, as well as venues for culture, sports and social activities, had profound impacts on lifestyles during the lockdown. As a result, the reasons, contexts, and ways of consuming may have changed. Indeed, alcohol is a prominent feature at social events such as parties and at certain venues such as bars and restaurants. For many consumers, the reasons for drinking are linked to both the pleasure procured by the product itself (taste, relaxation, disinhibition, anxiolytic effect) and its social aspect [13,14]. The reasons for smoking vary according to the profile of the smokers and their level of consumption. While some smoke in social situations with very little ritual attached, the consumption habits of the majority are very much rooted in their daily lives. Cigarettes are also sometimes associated with pleasure and conviviality. They can also be used in response to anxiety, to fill a void, manage emotions or concentrate, and are sometimes linked to a sense of identity and belonging to a group [15,16,17]. Physical, psychological and behavioural dependence can develop.

In the first weeks of the health crisis, changes regarding substance use were observed in several countries. The pandemic and the different measures implemented seemed to have an unfavourable impact on tobacco and alcohol consumption [18,19,20]. Given this context, an increase in tobacco and alcohol consumption within France was a conceivable risk. From the very beginning of the COVID-19 pandemic, Santé publique France, the national public health agency, set up the CoviPrev and ViQuoP surveys—the first quantitative, the second qualitative—in order to monitor the behaviours and mental health of the population in the context of the health crisis. These two surveys also addressed changes in tobacco and alcohol consumption. Based on a mixed methodology, this article presents a secondary analysis of these surveys, supplementing the first analyses performed at the very beginning of the lockdown [21]. It aims to describe the changes in self-reported tobacco and alcohol consumption during the first lockdown (April 2020) and the gradual lifting of the lockdown (May and June 2020), to identify factors associated with those changes, and to explore changes in the reasons for consumption.

## 2. Materials and Methods

### 2.1. Coviprev Study Design

The CoviPrev quantitative survey was a repeated cross-sectional online survey of the adult population conducted on an access panel constituted by specialists from the research firm BVA, who also helped draw up the questionnaire and collected the data. The survey was launched in the week of 23 March 2020, with successive weekly waves concerning a sample of 2000 people aged 18 years and over. The aim was to monitor a set of indicators related to health behaviours, fears and opinions specifically associated with COVID-19 and the lockdown. The samples were built using the quota method to represent the distribution of sex, age, socio-professional category, size of the urban area and region as found in the French population. The estimations were adjusted to the structure observed in the 2016 INSEE population census.

#### 2.1.1. Data Collection

Consumption of tobacco and alcohol was measured in Wave 2 (collection from 30 March to 1 April), Wave 5 (28 April to 30 April), Wave 7 (13 May to 15 May), Wave 8 (18 May to 20 May) and Wave 11 (22 June to 24 June). Due to the changing constraints related to the duration of the interviews, the information collected differs slightly between these waves.

#### 2.1.2. Variables

Respondents who reported smoking “cigarettes, including rolled cigarettes” or “only other types of tobacco (a pipe, cigar, shisha…)” at the time of the survey were considered current smokers.

Changes in tobacco consumption among current smokers were measured using the following question: “Compared to before lockdown (*Waves 2 and 5*)/Compared to February 2020 (before lockdown began, *Wave 7 onwards*), how has your tobacco consumption changed?” with three response options: “it has increased/it has remained stable/it has decreased”. Those who declared having stopped smoking during lockdown were only identified from Wave 5.

For alcohol, the question directly addressed changes in behaviour: “Compared to before lockdown/Compared to February 2020 (before lockdown began), how has your consumption of alcoholic beverages changed, concerning beer, wine, cider, spirits, champagne or any other type of alcohol, even when low in alcohol?” with four response options: “it has increased/it has remained stable/it has decreased/I never drink alcohol”. Those who did not select the last option were therefore considered as “drinkers”.

Those who had increased/decreased their consumption were asked about the reasons why (*Waves 2 and/or 5*): ‘for what reason(s) has your tobacco consumption increased (*Waves 2 and 5*)/decreased (*Wave 5*)? (several answers possible); and “what is the main reason why your alcohol consumption has increased (one answer possible for “main:” reason)/for what reason(s) has it decreased?” (several answers possible). Closed response options were proposed. The Coviprev questions are provided in the Appendix A.

The following sociodemographic variables were collected: sex, age, level of education, perceived financial situation, working conditions (remote, unchanged…), being the parent of a child (or children) under 16 years of age, living alone or with others. Anxiety and depression were measured using the Hospital Anxiety and Depression Scale (HADS) [22]. This scale was translated and validated in French by Lepine JP et al. [23]. Its psychometric properties are considered reliable for the identification of anxiety symptoms in general and clinical populations [24,25].

#### 2.1.3. Analysis

The survey waves were grouped by month. The differences between the changes in reported consumption were tested statistically using Pearson’s Chi-squared test between the waves of April (Waves 2 and 5) and those of May (Waves 7 and 8), and between the waves of May (Waves 7 and 8) and that of June (Wave 11). The differences in changes between the April and June waves were not analysed because some of the samples were not independent (n = 143 respondents common to Waves 2 and 11, n = 206 respondents common to Waves 5 and 11–the identification of repeat respondents was possible as the sample had been recruited from an access panel).

The reported changes in tobacco consumption (increase/stability/decrease) were estimated for each wave among those who declared themselves as current smokers at the time they were surveyed.

Multinomial regressions were conducted to identify the factors associated with changes in tobacco and alcohol consumption (increase and decrease) compared to stable consumption for the waves of April (n = 817 smokers and n = 2708 drinkers) and May (n = 782 and n = 2800, respectively). The analyses were conducted separately for each of the two periods because the health and social contexts were different (strict lockdown in April and gradual lifting of lockdown in May). The June wave was excluded from the analysis due to the smaller group size. All variables that were significantly related to changes in tobacco or alcohol consumption in the bivariate analysis (for at least one wave) were introduced into the final regression models.

### 2.2. ViQuop Study Design

The qualitative survey ViQuoP (for *Vie Quotidienne et Prévention* [daily life and prevention]) was performed on a group of 60 participants aged between 19 and 73 years who were interviewed on a regular basis about diverse and varying health topics. ViQuoP was conducted through written exchanges via an online platform run by the Kantar Public institute. The panel was constituted to include a diversity of socioeconomic situations (sex, age, socio-professional category, household composition, living environment: urban/rural, outside space (garden, balcony, etc.)/no outside space, Paris region/other regions). The description of the sample is presented in a dedicated document [26]. The study started on 30 March 2020 and lasted for three months. The participants were contacted on 18 occasions regarding their experiences during the crisis period (mental health, diet, physical activity, use of psychoactive products, emotional and sexual relationships, social relationships), their prevention practices with regard to COVID-19 (handwashing, adoption of protective measures, isolation and screening) and their opinions on prevention-related communication materials produced by Santé publique France. The participants were paid 250 euros for the entire study.

#### 2.2.1. Data Collection

On two occasions, the participants answered questions (see Appendix A) regarding their consumption of psychoactive products (alcohol, tobacco, cannabis). In the fourth week of lockdown (7–8 April), the questions concerned perceived changes in their use of psychoactive products and the reasons for those changes. Questions about mental health were also asked on this occasion. Two weeks into the post-lockdown period (20–25 May), the participants were again asked about the changes in their consumption and also about their opinion on a smoking cessation campaign. The questions were open-ended, and the participants provided personal written responses that were not shared among the group.

#### 2.2.2. Analysis

Psychosociologists at Kantar Public performed an initial read-across of the written responses obtained from each of the two occasions that the participants were asked about consumption. Thematic categories [27] were identified in order to produce an initial analysis. The material was then compiled and reanalysed in order to verify the categories and apply a longitudinal perspective. The participants used pseudonyms for identification purposes, thereby ensuring their anonymity.

## 3. Results

The sample is described in Table 1. Around one in five people declared themselves as smokers at the time of interview for the CoviPrev survey (21% in April, 20% in May, 22% in June). Less than one-third of respondents declared that they never drank alcohol, and around seven in 10 people declared drinking alcohol (68% in April, 70% in May and 72% in June).

### 3.1. Changes in Consumption

#### 3.1.1. Changes in Tobacco Consumption

The proportion of smokers who reported an increase in their tobacco consumption compared to before lockdown did not change significantly from month to month: between 27% and 32% depending on the survey period (Table 2). The proportion of smokers who reported having reduced their tobacco consumption decreased from the start of the post-lockdown period: 17% of smokers in April versus 12% in May (*p* = 0.02), with no significant change thereafter (10% in June). The proportion of smokers with the same consumption as before lockdown remained steady between the different periods.

#### 3.1.2. Changes in Alcohol Consumption

The proportion of drinkers who reported an increase in their alcohol consumption remained steady between April (13%) and May (13%), and then decreased slightly in June (10%, *p* < 0.001) (Table 2). However, there was an increase during the lockdown, between the two April waves, with a higher proportion of people reporting increased consumption at the end of April compared to the beginning of April (16% vs. 11%, *p* < 0.001). The proportion of those who declared the same consumption as before the lockdown increased with the gradual lifting of the lockdown: 68% in June versus 63% in May (*p* < 0.01) and 62% in April. Just under one-quarter of people said they had reduced their consumption compared to before the lockdown in April (25%) and May (24%), and around one in five in June (22%).

#### 3.1.3. Contrasting Behaviours and Changes Reported by the ViQuoP Respondents

These trends resonate with the accounts of the ViQuoP qualitative survey respondents, including 25 smokers and 40 drinkers. Two behaviours were reported starting from the first time the respondents were contacted (7 April 2020), with some declaring that they had control over their consumption (stability, decrease or even cessation of consumption of a product), while others had increased their consumption of at least one product in a more or less controlled way. Changes in consumption patterns were also reported: different types of alcohol consumed, sometimes more frequently but in a lower overall quantity, for example. When asked again in mid-May 2020, some people who had increased their consumption identified phases, indicating an increase during the first few weeks of lockdown, followed by a slowdown then, after a few weeks, a regulation of consumption among those who had tried and succeeded to regain control.

“*I had noticed that I was smoking more at the beginning of lockdown. I took drastic action and was able to bring my tobacco consumption quickly under control. I think it happened because I was stressed with being stuck at home. […] After the lockdown, I have not felt the need to consume more tobacco.*”

Male, >60 years, upper socio-professional category, living alone outside the Paris region

During the first few days post-lockdown, the respondents did not report a significant increase in their consumption, particularly as the respect of social distancing severely limited the number of gatherings of friends and family and, therefore, the opportunities for consumption in a social setting. For some people, however, the lifting of the lockdown led to celebratory contexts that resulted in increased alcohol consumption (more rarely for tobacco). After a few days of post-lockdown, a return to a more or less ‘normal’ state was reported—especially for those resuming work outside the home or having returned to their usual dwelling. This return to normal was reflected by an increase or decrease in consumption depending on consumption habits prior to the lockdown and those acquired during it.

### 3.2. Reasons for Changes in Consumption

When contacted on 7 April 2020 for the ViQuoP study, those who had increased their consumption of tobacco or alcohol mentioned the need to compensate for boredom, sometimes caused by not working. For some, after-work/early-evening get-togethers had become a moment of heightened sociability. During lockdown or not, this type of occasion is very often associated with alcohol. As a result, those occurring virtually from behind a computer, or within a household group, added a new dimension and sometimes a new frequency to such drinking occasions.

“*[During lockdown] when it came to alcohol, tobacco and cannabis, my consumption increased. I smoked a lot more tobacco because I was bored, the same with cannabis; and as for alcohol, I organised virtual get-togethers with my friends almost every night (with different groups), which meant we had a drink together, whereas I do not normally go to bars every night…*”

Male, 21 years, lower socio-professional category, living with several adults outside the Paris region (suburban, no outdoor space)

The stress and anxiety generated by the health and social situation, by its intense media coverage, but also the frustration engendered by the restrictions were mentioned as reasons behind their increased consumption. Sometimes it was about allowing oneself some form of pleasure in an exceptional and difficult situation.

“*For alcohol, I rarely had any at home because I used to drink it in a festive context outside my home. And I had stopped smoking cigarettes completely. […] I am obliged to buy alcohol and have it at home. And I have started smoking again after stopping for almost three months. I have decided not to cut down because the situation is already very difficult on a day to day basis. It is somewhat burdensome and alcohol enables you to detach a little. And cigarettes offer an additional moment of relaxation. And what with already being deprived of our freedom of movement, this would only add to the restrictions on our freedoms. And this is not something I want.*”

Female, 43 years, upper socio-professional category, living with a young adult son outside the Paris region

“*I tend to smoke a bit more. This is generated by the fact that we get bored more with nothing to do, especially in view of the current situation which seems complicated. No matter what anyone says, the media is reporting horrendous figures concerning people who are sick and above all the deaths that are increasing daily. This inevitably leads to a situation of permanent stress.*”

Male, 61 years, upper socio-professional category, living alone outside the Paris region

Remote working was mentioned as a situation that could promote consumption, by increasing the stress caused by the pace of work and giving people the possibility to consume in front of the screen.

“*When I’m teleworking, which is definitely not for me, I get bored and stressed, so I smoke. If I’m working from home it’s 15 cigarettes a day, in the office it’s 5 cigarettes, so there is a big difference*”

Female, 28 years, middle socio-professional category, living alone outside the Paris region

For those who had decreased or stabilised their consumption, three attitudes are noted. Firstly, some people reported that the situation had little impact on their mood and lifestyle (they continued to work outside the home or were elderly, for example), and as such made little change to their consumption, which was often quite low.

“*As for alcohol consumption, I drink in the evening after my working day, which hasn’t changed because my job continued during lockdown.*”

Male, 50 years, upper socio-professional category, living alone outside the Paris region

Others expressed a desire to limit their consumption, to ‘discipline themselves’ because they quickly identified the potential risks of such a period.

“*Before, I used to drink wine when I went out, or with friends; now we have a WhatsApp—meeting every evening, which encourages me to have a drink every evening, but I have slowed down because I don’t want it to become a habit.*”

Female, 73 years, upper socio-professional category, retired, living alone in the Paris region

Finally, for others, the lockdown restrictions provided an opportunity to reduce consumption or were even seen as a blessing in this sense, particularly among those with the lowest levels of consumption.

“*During the lockdown, I allowed myself a beer or two on some evenings to mark the end of the day and try to have a little fun. However, as I did not go out and I was alone, I did not have any evenings of heavy drinking. Overall, I think I consumed less or as much alcohol as usual.*”

Male, 39 years, middle socio-professional category, living alone in the Paris region

Consumption habits within the home were sometimes maintained. The presence of non-smokers and children in the home may have limited tobacco consumption because the desire to avoid passive smoking meant that it was only possible outdoors. Similarly, people who did not usually drink at home made little change to that habit. Previous consumption centred mainly around a social context of going out (bars, restaurants), which was no longer possible, was not always compensated for by new forms of consumption in the home, whether for alcohol or ‘social’ cigarette smoking. Finally, in a smaller number of cases, difficulties in obtaining provisions (opening hours, fear of infection) were reported, with some people deciding not to go shopping for alcohol and tobacco.

“*I’m not allowed to smoke in the flat which helps me a lot to cut down. I could go out into my yard to smoke but I hold back, telling myself it’s not worth it.*”

Female, 21 years old, student, living with several adults in the Paris region

“*I used to smoke 4/5 cigarettes a day at convenient opportunities like during coffee breaks, on the terrace with friends, during after-work drinks and in the evening… “Relaxation” cigarettes! On the second day of the lockdown I ran out of cigarettes and never any around… I refused to go to the shop for a purchase so contradictory to the health measures. I stopped smoking, I don’t feel the need to*”

Female, 67 years old, upper socio-professional category (retired), living with spouse and two adult children in a rural region

The results of the April waves of CoviPrev (Table 3) confirm and rank the reasons for the changes presented above. They show that among those smokers having increased their tobacco consumption in April (Waves 2 and 5), the main reasons given were boredom and lack of activity (74%), stress (52%), followed by difficulties in obtaining smoking cessation aids (13%), pleasure (12%) and other reasons (10%) which include, for example, the conditions of remote working. For alcohol, the reasons were mainly pleasure (49%) and boredom (30%), with stress accounting for a much smaller proportion (13%). No significant changes were observed between the two April waves.

The main reasons for the decrease in consumption in Wave 5 of the survey also differed depending on the products (Table 3). For tobacco, among those having reduced or stopped smoking during the lockdown, health reasons were often given: for one’s health (41%), for one’s physical condition (25%), or for the health of others in the household (16%). In addition, the period and its restrictions were seen by some as a voluntary opportunity or a constraint to cut down or stop smoking: 43% of the smokers said that it was a good time for them and 31% said that the reduction in social occasions was a reason for reducing their tobacco consumption. Finally, non-consumption habits at home were mentioned by 38% of smokers. Regarding alcohol, three quarters stated that it was the absence of social activities that affected their consumption (75%). Consumption habits at home (23%) and health reasons (18%) were also mentioned.

### 3.3. Factors Associated with Increased or Decreased Tobacco and Alcohol Consumption

The factors associated (Table 4) with a reported increase or decrease in tobacco consumption versus stable consumption changed depending on the wave of the survey. Among the smokers, during the April lockdown period, being a woman, having a post-secondary qualification, not working or being unemployed, or working at home (compared to working outside the home), as well as having high levels of anxiety or depression, were independently associated with an increase in smoking; being 65 years of age or older appeared to be protective. In May, only poor mental health remained associated with increased tobacco consumption, while being 65 years of age or older remained protective.

Regarding a decrease in smoking, in April, having an upper or post-secondary qualification was likewise associated with decreased consumption, whereas no factors were significantly associated with the decrease observed in May.

Regarding alcohol consumption (Table 5), the factors associated with an increase also differed depending on the wave of the survey. In April, being under 35 years of age, living in a large urban area, being the parent of a child aged 16 or under, and having high levels of anxiety or depression were associated with an increase in alcohol consumption versus stable consumption. Declaring oneself as “just getting by” financially was negatively associated with an increase in alcohol consumption. In May, age also appeared to be protective (50 years or older), while poor mental health (high levels of anxiety or depression) was associated with increased alcohol consumption.

Factors associated with the decrease in alcohol consumption in April were being under 35 years of age, not working or being unemployed during this period, living alone and living in a large urban area. In May, only age remained associated with a decrease, but reporting depression appeared to be protective.

## 4. Discussion

The analysis of CoviPrev and ViQuoP confirms that the March–April 2020 lockdown period and then the start of the gradual lifting of lockdown from 11 May 2020 in France may have limited tobacco and alcohol consumption for some and, on the contrary, favoured it for others.

The increases in tobacco and alcohol consumption concerned between one-quarter and one-third of smokers according to the periods studied, as well as one-tenth of drinkers. Boredom and stress for smokers, and boredom and pleasure-seeking for drinkers, were the main reasons reported to explain these increases during the strict lockdown period. Increases were more frequent for certain profiles, such as women for tobacco (in April) and young people for alcohol, as well as people with poor mental health (anxiety and depression), irrespective of the period and for both products.

Studies in other countries also identified higher-risk profiles and a strong link between mental health and increased tobacco [18,28] or alcohol consumption [29,30,31,32] during the lockdown. The profiles identified and reasons reported for the increase showed similarities with our results. For instance, stress was associated with both increased and decreased smoking among Dutch smokers [28]. In Belgium, younger age, children at home, or being furloughed were associated with an increase in alcohol consumption. Boredom, lack of social contact, loss of daily structure, an after-work reward, loneliness or conviviality were the main reasons reported by smokers and drinkers for increased consumption [33]. In addition, many studies point to links between economic situation, mental health and psychoactive substance use [5,6,7,8,9,10,11,34,35,36]. The health crisis had an impact on the financial situation of one-quarter of the French population as far back as May, affecting mainly those whose initial standard of living was already low, those with children, and young people [37]. According to the French Coclico survey, women and people with the poor financial situation were particularly at risk of psychological distress during the first lockdown [12]. The economic effects of the crisis and the stress linked to the epidemic itself could therefore explain why our study found that young people, women and those out of work were more vulnerable to increases in consumption.

The results of CoviPrev and ViQuoP surveys suggest that people whose lifestyles were strongly modified more frequently increased their consumption of tobacco or alcohol in April. These modifications may have been related to their professional situation, especially for young people and the more socially advantaged, with remote work possibly facilitating smoking during ‘office’ hours, or inactivity leading to boredom. They may also have been related to living conditions: the presence of a child (or children) under 16 years of age mostly kept at home due to school closures, or urban dwellers who may have had less outdoor space.

The Epicov study [38] shows that working from home was highly commonplace during lockdown for executive staff but much more limited for non-executive staff and key workers who continued to work outside the home. Young people and those in the most precarious situations were more affected by furlough schemes and lay-offs. Furthermore, a later French survey of employees, conducted in September 2020, shows that the main reported reason for the increase in psychoactive substance use was working conditions (isolation from colleagues, workload, and changes of tasks) [39]. In the Coclico study, people with low levels of social support and those confined to over-occupied housing were identified as being particularly at risk of psychological distress [12]. The daily lives of some profiles were, therefore, particularly disrupted, encouraging the consumption of tobacco and alcohol during the lockdown. In May, the easing of restrictions with the gradual reopening of schools, the possibility to travel and the resumption of some onsite work activities could explain why these factors are no longer associated with an increase in consumption from the start of the post-lockdown period.

The analyses also show stable consumption for the majority of the respondents, and that certain behaviours were more favourable to health for some people during this period, primarily for alcohol: 22% to 25% of drinkers and 10% to 19% of smokers declared having reduced their consumption, depending on the wave. This may have been because of a desire to control it for reasons of health and well-being, especially observed in the case of tobacco, or because of external constraints that modified consumption habits, especially observed for alcohol. These findings are consistent with those made in other countries [28,31]. In addition, some studies show an increase in motivation and attempts to stop smoking due to the fear of developing a severe form of COVID-19 [40,41,42].

However, certain profiles are associated with both increased and decreased consumption, suggesting heterogeneous behaviours within the same population segments. Thus, the youngest people more often both increased and decreased their alcohol consumption in April. The same phenomenon was observed for smokers with the highest level of education in April, people living in a large urban area for alcohol in April and people with high levels of depression for alcohol in May. These findings are consistent with the hypothesis that the epidemic and lockdown had differentiated effects on behaviour according to lifestyle, consumption habits and the impact of the COVID-19 crisis on personal life [28,43].

Any changes observed over the three months of the survey were fairly limited, albeit with a tendency for a reduction in the proportion of people declaring a decrease in their tobacco consumption from May onwards, and a reduction in those declaring an increase in their alcohol consumption from June onwards—which could indicate a return to usual consumption habits starting from the time that lockdown was lifted. In June, when the restrictions were widely lifted, a significant proportion of smokers and drinkers (28% and 10%, respectively) reported higher consumption than before the lockdown, suggesting that these unhealthy behaviours could persist.

In France, as in other countries, different studies note this dual movement of increasing and decreasing consumption during lockdown. Often, an increase in tobacco consumption was more frequent than a decrease, [28,44] and inversely for alcohol [45,46,47]. Nevertheless, in some studies, particularly in English-speaking countries, the reported increases in alcohol consumption can largely outweigh any parallel decrease [23,24,42], whereas more favourable trends are observed for tobacco [40,42]. These findings could be due to cultural differences such as the lower prevalence of smokers in English-speaking countries compared to France, or the contrasting drinking habits (binge drinking remains common in English countries, whereas the French tend to drink lower quantities more regularly). The varying strictness of policies to control the epidemic (i.e., in some countries, the purchase of alcohol beverages was prohibited during the lockdown) or the resulting obstacles to procuring these products could also influence outcomes for consumption. Likewise, the periods studied within lockdown or differences in survey methods (probabilistic or non-probabilistic survey, transversal or longitudinal, interview period, etc.) could have an impact on the results.

The data analysed in this article have limitations. On the one hand, the CoviPrev sample size was small, preventing extrapolation to the general population (for smoking cessation in particular). The study is based on non-probabilistic samples obtained through an online panel, limiting their representativity. The proportion of people with a high level of education was thus higher than in the general population; also, the prevalence of smoking was lower, and alcohol abstention higher than levels measured in French representative studies [48,49]. Comparisons between the April and June waves were impossible due to some respondents being common to several waves. Moreover, desirability and memory biases may have existed insofar as people had to recall their pre-lockdown consumption several weeks later and then compare and self-report any changes. In addition, data regarding the level of consumption were not available for all waves; thus, it was not possible to introduce this aspect to the analysis or to explore the impact of consumption habits. Finally, a causal link with lockdown, the epidemic, or both, cannot be fully established, especially for smoking cessation rates, given that people also quit in a usual context.

On the other hand, ViQuoP was a qualitative study based on a small sample of psychoactive product users, comprised of profiles with specific characteristics (panel with Internet access and comfortable with reading/writing), which limits the generalisation of the results. Furthermore, the questions asked were open-ended, but only one follow-up interview was possible given the time-frame and as stipulated in the protocol, which did not allow for an in-depth and detailed description of all situations. Repeatedly asking people about their health in general and their consumption in particular may have affected their attitudes and consumption.

Finally, and as in most general population surveys, the most precarious and vulnerable populations (i.e., disadvantaged groups, people experiencing homeless, etc.) were not represented in the sample because they were not covered by the sampling methods.

## 5. Conclusions

Despite these limitations and thanks to the triangulation of the quantitative and qualitative data, these highly responsive surveys have made it possible to measure trends in tobacco and alcohol consumption in the general population during the first lockdown in France, the profiles associated to these changes and the experiences of smokers and drinkers. The results of these surveys show the necessity and acceptability [26] of the prevention campaigns disseminated during the COVID-19 epidemic period to encourage and support smoking cessation or lower alcohol consumption. Messages should be tailored to the situation [50] and can promote remote assistance tools, which are particularly useful during periods of travel restriction. Special attention for the highest risk profiles is necessary.

## Figures and Tables

**Table 1:** Sociodemographic characteristics, anxiety and depression levels, smoking status and alcohol use, in each wave.

	Wave 2 (n = 2003) 30 March–1 April 2020		Wave 5 (n = 2000) 28–30 April 2020		Wave 7 (n = 2000) 13–15 May 2020		Wave 8 (n = 2000) 18–20 May 2020		Wave 11 (n = 2000) 22–24 June 2020	
	Unweighted n	Weighted %	Unweighted n	Weighted %	Unweighted n	Weighted %	Unweighted n	Weighted %	Unweighted n	Weighted %
**Sex**										
Male	954	47.6	908	47.6	918	47.6	907	47.6	907	47.6
Female	1049	52.4	1092	52.4	1082	52.4	1093	52.4	1093	52.4
**Age group**										
18–34 years	467	25.6	459	25.6	442	25.6	469	25.6	469	25.6
35–49 years	533	25.5	529	25.5	506	25.5	527	25.5	525	25.5
50–64 years	523	25.1	523	25.1	536	25.1	515	25.1	518	25.1
65+ years	480	23.8	489	23.8	516	23.8	489	23.8	488	23.8
**Size of urban area**										
<100,000 inhabitants	1071	53.5	1069	53.5	1068	53.5	1053	53.5	1036	53.5
≥100,000 inhabitants	932	46.5	931	46.5	932	46.5	947	46.5	964	46.5
**Living alone**										
No	1540	76.9	1534	76.9	1558	78.3	1561	78.3	1553	78.2
Yes	463	23.1	466	23.1	442	21.7	439	21.7	447	21.8
**Parent of children aged 16 or under**										
No	1417	71.0	1449	72.2	1464	72.4	1418	71.0	1411	70.5
Yes	586	29.0	551	27.8	536	27.6	582	29.0	589	29.5
**Education level**										
Less than highschool graduate	600	29.9	561	28.7	533	27.9	588	29.7	576	29.4
High school graduate	509	25.7	493	24.6	474	24.2	490	24.7	471	23.6
College graduate	894	44.4	946	46.7	993	47.9	922	45.6	953	47.0
**Perceived financial situation**										
Difficult financial situation	388	19.7	363	18.7	348	17.9	378	19.4	367	18.4
Need to be careful	562	28.2	517	26.1	538	27.2	533	26.5	563	28.1
Good financial situation	1053	52.1	1120	55.2	1114	54.83	1089	54.1	1070	53.6
**Working conditions**										
Not working (inactive, unemployed, furloughed, on leave)	1325	66.8	1268	64.1	1083	54.3	1062	54.1	992	51.2
Working from home	315	15.4	324	15.7	317	14.8	277	13.3	166	7.8
Working outside the home	363	17.8	408	20.3	600	30.9	661	32.7	842	41.0
**Anxiety level (HADS)**										
Normal (0–7)	1121	55.8	1265	62.6	1246	62.2	1273	63.9	1348	67.5
Possible (8–10)	452	22.7	380	19.3	407	20.3	386	19.2	349	17.4
Probable (11–21)	430	21.5	355	18.1	347	17.6	341	16.9	303	15.1
**Depressive mood (HADS)**										
Normal (0–7)	1152	57.6	1175	58.4	1345	67.1	1373	68.9	1451	72.4
Possible (8–10)	452	22.6	448	22.3	372	18.8	380	19.0	331	16.4
Probable (11–21)	399	19.9	377	19.3	283	14.1	247	12.1	218	11.2
**Smoking status**										
Smoker	422	21.2	395	20.2	379	19.9	403	20.6	434	21.7
Non-smoker	1581	78.8	1605	79.8	1621	80.1	1597	79.5	1566	78.3
**Alcohol use**										
No alcohol use	659	33.3	636	31.7	630	31.7	570	28.6	569	28.3
Alcohol use	1344	66.7	1364	68.3	1370	68.3	1430	71.4	1431	71.7

HADS: Hospital Anxiety and Depression Scale.

**Table 2:** Changes in tobacco and alcohol consumption reported over the five waves from April to June 2020: proportion of smokers and drinkers who increased, maintained or decreased their consumption at each wave. Comparisons between waves of the same month and waves pooled by month.

	Lockdown (April)							Gradual Lifting of Lockdown (May and June)										Comparisons of the Waves	
Data Collection Period and Survey Waves	April Waves (W2 and W5) n = 4003			W2 30 March–1 April		W5 28 April–30 April		May Waves (W7 and W8) n = 4000			W7 13 May–15 May		W8 18 May–20 May		June Wave (W11) 22 June–24 June n = 2000			AprilW/MayW	MayW/JuneW
				W2/W5: *p* = ns							W7/W8: *p* = ns				W7/W11: *p* = ns W8/W11: *p* = ns			*p* = 0.02	*p* = ns
**Tobacco** (among current smokers)	n	%	CI	n	%	n	%	n	%	CI	n	%	n	%	n	%	CI		
Increased	240	29.2	[26.2–32.5]	112	26.7	128	31.9	249	31.7	[28.4–35.1]	119	31.3	130	32.0	118	28.0	[23.8–32.5]		
Remained stable	442	54.3	[50.7–57.7]	231	54.7	211	53.8	442	56.7	[53.1–60.3]	217	58.0	225	55.5	273	62.2	[57.4–66.7]		
Decreased	135	16.5	[14.1–19.2]	79	18.6	56	14.3	91	11.6	[9.4–14.1]	43	10.7	48	12.4	43	9.8	[7.3–13.0]		
Total	817			422		395		782			379		403		434				
**Smoking cessation during lockdown**				-		23		43			23		20		34				
				W2/W5: *p* < 0.001							W7/W8: ns				W7/W11: *p* < 0.001 W8/W11: *p* = 0.02			*p* = ns	*p* < 0.001
**Alcohol** (among drinkers)	n	%	CI	n	%	n	%	n	%	CI	n	%	n	%	n	%	CI		
Increased	354	13.3	[12.0–14.7]	143	10.7	211	15.9	369	13.2	[11.9–14.5]	185	13.6	184	12.8	145	10.2	[8.6–11.8]		
Remained stable	1686	61.8	[59.9–63.6]	878	64.8	808	58.9	1759	62.7	[60.8–64.4]	849	61.6	910	63.7	980	68.3	[65.8–70.7]		
Decreased	668	24.9	[23.2–26.5]	323	24.4	345	25.3	672	24.1	[22.5–25.8]	336	24.8	336	23.5	306	21.5	[19.4–23.7]		
Total	2708			1344		1364		2800			1370		1430		1431				

CI: 95% confidence interval; W2/W5 *p*-value: Pearson’s chi-squared test between consumption reported in Wave 2 and that reported in Wave 5; AprilW/MayW *p*-value: Pearson’s chi-squared test between the evolution of consumption reported in April (waves 2 and 5 together) and that reported in May (waves 7 and 8 together); ns = non-significant.

**Table 3:** Reasons reported for the increase (Waves 2 and 5), decrease or cessation (Wave 5) of tobacco and alcohol consumption.

Reasons Reported for the Increase ^1^ in Tobacco and Alcohol Consumption in April (Waves 2 and 5)	Tobacco n = 240		Alcohol n = 354	
	n	%	n	%
Boredom, lack of activity	176	73.7	107	30.2
Stress	127	52.5	48	13.1
Pleasure	29	12.3	173	48.9
Difficulty obtaining ^2^ smoking cessation aids (nicotine replacement treatments, e-cigarettes) or psychological support	30	12.6	-	-
Another reason	23	9.7	26	7.8
**Reasons reported for the decrease or cessation of tobacco ^3^ and alcohol consumption in Wave 5**	**Tobacco n = 79**		**Alcohol n = 345**	
	n	%	n	%
I avoid smoking/drinking in my home	30	37.8	78	23.3
For the health of the people who live with me	12	15.9	-	-
For my health	34	41.4	63	18.1
To get fit or stay in good physical condition	19	25.1	42	12.4
It is a good time to cut down or stop smoking/drinking	33	42.6	38	11.2
I have difficulties obtaining cigarettes or tobacco/alcohol	18	22.9	12	3.9
Fewer social or festive occasions, opportunities to smoke/drink with friends or colleagues	26	31.5	261	75.4
I am less stressed than usual	11	14.1	15	4.6
Another reason	9	11.0	22	6.6

^1^ respectively among smokers and drinkers who reported an increase in Wave 2 or 5; ^2^ pools the following response options: ‘I am on replacement treatment (patches, gum…) or another treatment (Champix, Zyban) that I had to interrupt’, ‘I use an electronic cigarette and I have no liquid left to refill it or it no longer works’ and ‘The psychological support I was receiving is no longer possible’; ^3^ among those who had cut down and those who had stopped smoking during the lockdown. The columns add up to more than 100% because in some cases, more than one answer was possible.

**Table 4:** Bivariate analyses between sociodemographic and mental health variables, and changes in tobacco consumption compared with before the lockdown as reported in the April and May waves (row percentages). Adjusted relative risk ratios and confidence intervals from multinomial logistic regressions (increase and decrease vs. stable consumption).

	April Wave (W2 and W5); n = 817										May Wave (W7 and W8); n = 782									
			Increased				Decreased						Increased				Decreased			
	Unweighted n	Chi-2 *p*-Value	Weighted %	aRRR	CI	*p*-Value ^1^	Weighted %	aRRR	CI	*p*-Value ^1^	Unweighted n	Chi-2 *p*-Value	Weighted %	aRRR	CI	*p*-Value ^1^	Weighted %	aRRR	CI	*p*-Value ^1^
**Sex**		0.002										0.009								
Male (ref.)	394		23.8	1			16.1	1			350		26.2	1			13.0	1		
Female	423		34.6	1.62	[1.15–2.27]	0.005	16.9	1.16	[0.78–1.72]	0.459	432		36.6	1.41	[0.99–2.01]	0.051	10.3	0.82	[0.50–1.33]	0.435
**Age**		0.027										0.010								
18–34 years (ref.)	219		35.0	1			16.1	1			219		36.0	1			11.0	1		
35–49 years	273		31.8	0.95	[0.62–1.46]	0.835	17.5	1.08	[0.64–0.83]	0.757	250		33.0	0.88	[0.57–1.36]	0.588	8.5	0.63	[0.32–1.23]	0.180
50–64 years	241		23.3	0.67	[0.42–1.07]	0.096	14.8	0.85	[0.48–0.48]	0.571	223		31.5	1.02	[0.64–1.61]	0.926	13.0	0.99	[0.52–1.87]	0.981
65 years and over	84		20.0	0.50	[0.25–0.99]	0.047	19.3	0.83	[0.40–0.73]	0.630	90		15.0	0.43	[0.21–0.88]	0.022	19.0	0.94	[0.43–2.07]	0.889
**Working conditions**		0.026										ns								
Working outside the home (ref.)	195		22.2	1			15.7	1			291		32.3	1			9.2	1		
Not working (inactive, unemployed, furloughed, on leave)	501		29.7	1.55	[1.01–2.40]	0.046	16.9	1.31	[0.80–2.15]	0.271	391		30.7	1.11	[0.76–1.63]	0.571	13.4	1.43	[0.82–2.49]	0.199
Working from home	121		38.6	2.25	[1.28–3.94]	0.004	16.1	1.17	[0.59–2.31]	0.641	100		34.0	1.01	[0.59–1.74]	0.947	11.3	1.25	[0.57–2.73]	0.573
**Parent of a child aged 16 or under**		ns										0.015								
No (ref.)	532		27.0	1			17.1	1			512		28.8	1			13.6	1		
Yes	285		33.4	1.05	[0.72–1.55]	0.771	15.4	0.94	[0.59–1.50]	0.812	270		37.0	1.16	[0.80–1.68]	0.405	7.9	0.75	[0.42–1.34]	0.345
**Educational level**		0.002										ns								
<high-school graduate (ref.)	307		27.4	1			11.8	1			283		32.6	1			11.4	1		
high-school graduate	215		24.7	1.09	[0.70–1.69]	0.693	20.9	1.93	[1.16–3.22]	0.011	209		30.6	0.82	[0.53–1.26]	0.382	9.5	0.84	[0.45–1.57]	0.596
>high-school graduate	295		34.6	1.67	[1.11–2.50]	0.013	18.3	1.99	[1.21–3.30]	0.007	290		31.6	0.94	[0.62–1.42]	0.782	13.4	1.17	[0.68–2.03]	0.562
**Anxiety level (HADS)**		<0.001										<0.001								
Normal (0–7) (ref.)	428		20.9	1			18.6	1			435		21.7	1			12.7	1		
Possible (8–10)	188		33.2	1.56	[1.01–2.40]	0.041	13.1	0.79	[0.46–1.35]	0.399	169		33.0	1.64	[1.07–2.50]	0.021	13.0	1.41	[0.80–2.48]	0.228
Probable (11–21)	201		43.0	2.19	[1.38–3.47]	0.001	15.4	1.25	[0.70–2.22]	0.439	178		55.1	2.82	[1.81–4.39]	<0.001	7.3	1.13	[0.54–2.34]	0.742
**Depressive mood (HADS)**		<0.001										<0.001								
Normal (0–7) (ref.)	413		21.2	1			18.5	1			500		23.0	1			12.9	1		
Possible (8–10)	208		35.1	1.40	[0.91–2.16]	0.122	14.6	0.91	[0.53–1.54]	0.738	158		43.4	1.79	[1.17–2.73]	0.007	11.6	1.11	[0.60–2.06]	0.716
Probable (11–21)	196		39.8	1.77	[1.11–2.82]	0.015	16.5	1.04	[0.59–1.85]	0.875	124		51.1	1.89	[1.17–3.08]	0.009	6.2	0.69	[0.29–1.64]	0.408

HADS: Hospital Anxiety and Depression Scale; aRRR: adjusted relative risk ratio; CI: 95% confidence interval; ref.: multinomial logistic regression reference modality; Chi-2 *p*-value: *p*-value of the bivariate analysis between the changes in consumption (remained stable, increased, decreased) and the sociodemographic or mental health variables. ^1^ *p*-value: *p*-value of the relative risk ratio.

**Table 5:** Bivariate analyses between sociodemographic and mental health variables, and changes in alcohol consumption compared with before the lockdown as reported in the April and May waves (row percentages). Adjusted relative risk ratios and confidence intervals from multinomial logistic regressions (increase and decrease vs. stable consumption).

	April Wave (W2 and W5)										May Wave (W7 and W8)									
	n = 2708										n = 2800									
			Increased				Decreased						Increased				Decreased			
	Unweighted n	Chi-2	Weighted	aRRR	CI	*p*-Value ^1^	Weighted	aRRR	CI	*p*-Value ^1^	Unweighted n	Chi-2	Adjusted	aRRR	CI	*p*-Value ^1^	Weighted	aRRR	CI	*p*-Value ^1^
		***p*-Value ^1^**	**%**				**%**					***p*-Value**	**%**				**%**			
**Sex**		ns										<0.001								
Male (ref.)	1449		12.5	1			24.6	1			1445		11.4	1			22.8	1		
Female	1259		14.3	0.97	[0.76–1.24]	0.818	25.2	0.924	[0.76–1.11]	0.413	1355		15.2	1.01	[0.79–1.29]	0.927	25.7	0.96	[0.79–1.16]	0.718
**Age**		<0.001										<0.001								
18–34 years (ref.)	570		18.9	1			34.8				542		19.2	1			32.5	1		
35–49 years	716		18.8	0.7	[0.51–0.98]	0.032	20.4	0.46	[0.38–0.67]	<0.001	712		18.5	0.78		0.133	21.2	0.58	[0.43–0.77]	<0.001
50–64 years	721		10.2	0.46	[0.31–0.66]	<0.001	24.3	0.47	[0.42–0.73]	<0.001	779		10.9	0.5		<0.001	23.3	0.53	[0.40–0.70]	<0.001
65 years and over	701		5.9	0.23	0.13–0.33]	<0.001	20.9	0.3	[0.28–0.54]	<0.001	767		5.5	0.21		<0.001	20.8	0.38	[0.28–0.52]	<0.001
**Size of urban area**		0.001										0.016								
<100,000 inhabitants (ref.)	1454		12.3	1			22.7				1489		12.5	1			22.4	1		
≥100,000 inhabitants	1254		14.5	1.31	[1.0–1.65]	0.029	27.4	1.31	[1.04–1.53]	0.005	1311		14.0	1.1	[0.87–1.40]	0.85	26.2	1.17	[0.97–1.41]	0.087
**Working conditions**		<0.001										<0.001								
Working outside the home (ref.)	567		14.7	1		0.082	20.9	1			919		14.7	1			22.5	1		
Not working (inactive, unemployed, furloughed, on leave)	1689		11.6	1.32	[0.96–1.81]	0.082	26.5	1.63	[1.26–2.12]	<0.001	1457		10.8	1.25	[0.92–1.68]	0.142	24.6	1.26	[0.98–1.61]	0.061
Working from home	452		18.1	1.35	[0.93–1.96]	0.104	23.7	1.25	[0.9–1.72]	0.163	424		18.3	1.34	[0.96–1.87]	0.082	26.2	1.2	[0.90–1.60]	0.212
**Educational level**		0.046										0.041								
<high-school graduate (ref.)	713		10.8	1			23.2	1			751		10.1	1			23.4	1		
high-school graduate	672		13.3	1.23	[0.86–1.76]	0.242	26.7	1.09	[0.84–1.42]	0.487	611		14.3	1.23	[0.86–1.76]	0.239	23.7	1.01	[0.77–1.32]	0.925
>high-school graduate	1323		14.8	1.34	[0.97–1.85]	0.07	24.8	1.07	[0.84–1.36]	0.536	1438		14.4	1.36	[0.99–1.87]	0.055	24.8	1.07	[0.84–1.35]	0.553
**Lives alone**		0.009										0.043								
No (ref.)	2115		13.7	1			23.5	1			2178		13.6	1			23.1	1		
Yes	593		12.1	1.26	[0.91–1.74]	0.15	29.7	1.31	[1.05–1.65]	0.017	622		11.8	1.01	[0.73–1.36]	0.985	27.9	1.22	[0.98–1.53]	0.072
**Parent of a child aged 16 or under**		<0.001										<0.001								
No (ref.)	1953		10.4	1			26.3	1			2067		11	1			25.1	1		
Yes	755		20.8	1.54	[1.11–2.1]	0.006	21.1	0.88	[0.68–1.13]	0.335	733		19.3	1.16	[0.87–1.56]	0.299	21.5	0.85	[0.66–1.09]	0.209
**Perceived financial situation**		0.019										0.009								
Good financial situation (ref.)	1581		13.3	1			23.6	1			1616		11.9	1			23.4	1		
Just getting by, need to be careful	683		12.0	0.66	[0.48–0.89]	0.007	24.6	0.95	[0.76–1.20]	0.713	752		14	1.04	[0.78–1.38]	0.754	23.5	0.93	[0.75–1.17]	0.575
Difficult financial situation	444		15.5	0.89	[0.63–1.27]	0.525	29.7	1.27	[0.97–1.66]	0.079	432		16.7	1.08	[0.77–1.52]	0.635	27.8	1.12	[0.85–1.46]	0.411
**Anxiety level (HADS)**		<0.001										<0.001								
Normal (0–7) (ref.)	1660		9.8	1			25.5	1			1830		10.4	1			23.4	1		
Possible (8–10)	549		16.0	1.44	[1.02–1.93]	0.023	26.1	0.99	[0.77–1.27]	0.713	529		15.4	1.23	[0.90–1.68]	0.183	24.6	1.01	[0.79–1.29]	0.911
Probable (11–21)	499		21.9	1.63	[1.13–2.25]	0.005	21.3	0.8	[0.59–1.07]	0.14	441		22.4	1.68	[1.19–2.36]	0.003	26.4	1.05	[0.78–1.41]	0.736
**Depressive mood (HADS)**		<0.001										<0.001								
Normal (0–7) (ref.)	1626		9.7	1			24.0	1			1975		10.3	1			22.3	1		
Possible (8–10)	606		17.0	1.59	[1.19–2.21]	0.003	28.0	1.25	[0.99–1.59]	0.058	495		18.8	1.75	[1.28–2.39]	<0.001	28.8	1.51	[1.17–1.95]	0.001
Probable (11–21)	476		21.1	1.87	[1.39–2.81]	<0.001	23.7	1.12	[0.84–1.51]	0.418	330		22.4	2.02	[1.40–2.91]	<0.001	28.0	1.56	[1.13–2.13]	0.006

HADS: Hospital Anxiety and Depression Scale; aRRR adjusted relative risk ratio; CI: 95% confidence interval; ref.: multinomial logistic regression reference modality; Chi-2 *p*-value: *p*-value of the bivariate analysis between the changes in consumption (remained stable, increased, decreased) and the sociodemographic or mental health variables. ^1^ *p*-value: *p*-value of the relative risk ratio.

## Data Availability

The data presented in this study are available on request from the corresponding author.

## Appendix A

**Appendix A1. Tobacco and alcohol questions in the CoviPrev survey**
**Tobacco use**
Wave 2 1. **Are you currently smoking?** 1. Yes, cigarettes, including rolled cigarettes 2. Only other types of tobacco (pipe, cigar, shisha…) 3. No, I do not smoke Wave 5, Wave 7, Wave 8, Wave 11 1. **Are you currently smoking?** 1. Yes, cigarettes, including rolled cigarettes 2. Only other types of tobacco (pipe, cigar, shisha…) 3. No, I quit smoking during lockdown 4. No, I had quit before lockdown 5. No, I have never really smoked *If smoker* Wave 2, Wave 5 2. **Compared to before lockdown, how has your tobacco consumption changed?** Wave 7, Wave 8, Wave 11 2. **Compared to February 2020 (before lockdown began), how has your tobacco consumption changed?** 1. It has increased 2. It has remained stable 3. It has decreased Wave 2, Wave 5 *If smoker has increased consumption.* 3. **For what reason(s) has your tobacco consumption increased?** *Several answers possible. Random rotation of items except “another reason”.* 1. Stress 2. Boredom, lack of activity 3. Pleasure 4. I am on replacement treatment (patches, gum…) or another treatment (Champix, Zyban) that I had to interrupt 5. I use an electronic cigarette and I have no liquid to refill it, or it no longer works 6. The psychological support I was receiving is no longer available 7. Another reason Wave 5 *If smoker has decreased consumption.* 4. **For what reason(s) has your tobacco consumption decreased?** *If ex-smoker since the lockdown.* 4. **For what reason(s) did you quit smoking during the lockdown?** *Several answers possible. Random rotation of items except “another reason”.* 1. I avoid smoking in my home 2. For the health of the people who live with me 3. For my health 4. It is a good time to cut down or stop smoking 5. I am less stressed than usual 6. I have difficulties obtaining cigarettes or tobacco 7. Fewer social or festive occasions, opportunities to smoke with friends or colleagues 8. To get fit or stay in good physical condition 9. Another reason
**Alcohol consumption**
Wave 2, Wave 5 1. **Compared to before lockdown, how has your consumption of alcoholic beverages changed, whether concerning beer, wine, cider, spirits, champagne or any other type of alcohol, even when low in alcohol?** Wave 7, Wave 8, Wave 11 1. **Compared to February 2020 (before lockdown began), how has your consumption of alcoholic beverages changed, whether concerning beer, wine, cider, spirits, champagne or any other type of alcohol, even when low in alcohol?** 1. It has increased 2. It has remained stable 3. It has decreased 4. I never drink alcohol Wave 2, Wave 5 *If alcohol consumption increased* 2. **What is the main reason why your alcohol consumption has increased?** *Random rotation of items except “Another reason”.* 1. Stress 2. Boredom, lack of activity 3. Pleasure 4. Another reason Wave 5 *If alcohol consumption decreased* 3. **For what reason(s) has your alcohol consumption decreased?** *Several answers possible. Random rotation of items except “Another reason”.* 1. I avoid drinking in my home 2. For my health 3. It is a good time to reduce or stop drinking 4. I am less stressed than usual 5. I have difficulties obtaining alcohol 6. Fewer social or festive occasions, opportunities to drink with friends or colleagues 7. To get fit or stay in good physical condition 8. Another reason **Appendix A2. Substance use questions in the ViQuoP study.** First interrogation Now I would like to know a little more about your consumption of each of the following products: alcohol, tobacco, cannabis or other drugs. 1/ Which of these products did you use before lockdown or during lockdown, even occasionally? 2/ For each of the products that you used, has your consumption changed since lockdown began? If so, what has changed? This could be changes in quantity, frequency, type of product, way of consuming it (times, days, contexts, alone/with others), etc. 3/ For each of the products, what do you appreciate about this change in consumption? Do you consume for the same reasons as usual? Are there any other reasons? 4/ And conversely, for each of the products consumed, what negative elements/difficulties do you perceive concerning these new ways of consuming? Second interrogation We are going to talk about your consumption of substances such as alcohol, tobacco and cannabis. We had already discussed this topic on the platform at the beginning of April: today I would like to know how your consumption has evolved since our last discussion and what your feelings are about it. 1. Lockdown and consumption (alcohol, tobacco, cannabis, other substances). - How would you assess your consumption of these products during lockdown? Have you observed any changes in your consumption during the last month of lockdown, since our discussion on the subject? - How do you feel about your consumption and its evolution during lockdown? Is it positive? Negative? Why or why not? 2. End of lockdown and consumption - Since the gradual lifting of lockdown, has your consumption of these products changed? If so, how? And why, in your opinion?
